# Automation is not evidence of efficiency

**DOI:** 10.1186/s13750-026-00388-7

**Published:** 2026-07-02

**Authors:** Matthew Grainger, Sini Savilaakso, Neal Haddaway, Chris Pritchard

**Affiliations:** 1 Norwegian Institute for Nature Research, Torgarden, P.O. Box 5685, 7485 Trondheim, Norway; 2 Department of Forest Sciences, University of Helsinki, P.O. Box 66, 00014 Helsinki, Finland; 3 Freelance, Bangor, UK; 4 Institute of Health and Allied Professions, Nottingham Trent University, Derby Road, Mansfield, NG18 5BH UK

## Abstract

We welcome Hodgson et al. (2026) empirical evaluation of ontology-grounded large language models (LLMs) for data extraction in environmental evidence synthesis. The study makes a valuable contribution by quantifying performance across attribute types and by openly documenting where current approaches struggle.

However, we are concerned that framing the approach as having “the potential for LLMs to save some labour” in evidence synthesis may overstate the authors’ actual findings.

We welcome Hodgson et al. [1] empirical evaluation of ontology-grounded large language models (LLMs) for data extraction in environmental evidence synthesis. The study makes a valuable contribution by quantifying performance across attribute types and by openly documenting where current approaches struggle.

However, we are concerned that framing the approach as having “the potential for LLMs to save some labour” in evidence synthesis may overstate the authors’ actual findings. Across many synthesis-critical attributes (e.g. response variables, restoration actions, sampling and monitoring methods), their reported precision and recall are between 50 and 60%, with substantial false-positive and false-negative rates. At these levels, human effort is not avoided but displaced into the labour-intensive activities of result verification, correction, and reconstruction of extracted information.

This distinction matters. In evidence synthesis, labour costs in the data extraction stage are dominated not by the mechanical act of extraction, but by the cognitive work of interpretation, consistency checking, and error correction. Tools that perform poorly on interpretation-heavy fields may still be useful as assistive prompts or exploratory aids, but they do not yet justify claims of efficiency gains, particularly without direct evidence of reduced overall workload. More appropriately framed conclusions would distinguish between partial task automation and demonstrated reductions in total synthesis workload, which would require direct evaluation of verification time, correction burden, and overall human effort. Without estimates of human-human agreement, it is also difficult to contextualise whether observed AI-human disagreement reflects model limitations or task ambiguity, further complicating claims about automation.

The authors call for greater investment in ontology expansion and prompt engineering to improve extraction performance. While technically reasonable, this recommendation raises an important question of opportunity cost. Time spent refining ontologies and engineering prompts is time not spent conducting primary research, synthesising the literature, improving synthesis methods, or addressing substantive ecological questions. Before encouraging the research community to invest effort in optimising AI tools, there should be clearer evidence that such investment yields net gains in efficiency, reliability, or decision quality. Without such evidence, calls for further optimisation risk shifting the burden of tool development onto researchers rather than critically evaluating whether current AI approaches are sufficiently mature to justify environmental and economic costs of widespread adoption.

We emphasise that these concerns do not diminish the importance of the study. Rather, they highlight the need for greater precision in how their results are framed and avoiding overreach, particularly in abstracts that will shape downstream uptake. Framing such tools as requiring further development, rather than implying evidence of labour-saving benefits, would more accurately reflect the current evidence base and help avoid premature assumptions about efficiency in decision-relevant syntheses. Given the central importance of accuracy in evidence synthesis, these levels of disagreement warrant careful consideration before claims about efficiency gains are made.

As interest in AI-assisted evidence synthesis accelerates, careful alignment between empirical performance and headline claims will be of vital importance to ensure responsible, transparent, and trustworthy adoption. As the RAISE guidelines [2] say “claims about efficiency, labour saving, or acceleration should be *supported by evidence*, not inferred from task automation alone.”

## Data Availability

This manuscript does not report data generation or analysis.
